# Supplementary material for: Quantitative Characterisation of Low Abundant Yeast Mitochondrial Proteins Reveals Compensation for Haplo-Insufficiency in Different Environments
Source: Int J Mol Sci. 2022 Aug 1;23(15):8532. doi: 10.3390/ijms23158532 (PMC9369417; doi:10.3390/ijms23158532)
Supplement: Supplementary file 1 [file ijms-23-08532-s001.zip › ijms-1747393-supplementary.pdf]

Supplementary information

Transformed cells for GFP were subjected to confirmation PCR to verify the integration of the C-terminal tags at the 3' end of *FIS1* or *MDV1* ORFs. Confirmation primers were designed to amplify the upstream and downstream regions of the coding gene and the recombination site specific to the GFP-*His3MX6* cassette. For each strain, the reverse sequence for the upstream confirmation of the tag insert is specific to the cassette and is identical in both strain backgrounds. Similarly, the forward primer for the downstream confirmation of the histidine marker integration is kept the same for all strains. The GFP fusion was confirmed using the genomic DNA of the transformed colonies and the high fidelity LongAmp *Taq* DNA polymerase (New England Biolabs, UK) in a PCR reaction of 25µl. The confirmation primers designed for the *S. cerevisiae* fusion strains are listed in Table S1.

Haploid *S. cerevisiae* mating type was confirmed by colony PCR for the *MAT* locus. Confirmation primers of universal sequences were designed to amplify the *MAT*a and *MAT*α region in *S. cerevisiae* strains (Supplementary Table 1). Colonies of each strain background were suspended in 50µl sterile MilliQ water and heated at 95°C for 15 minutes, and 5µl of the cell suspension were used as template in a 25µl PCR reaction using the MyTaq Red DNA mix (Bioline, UK). PCR conditions were: 94°C for 1 minute, 54°C for 1 minute, 72°C for 1 minute and 72°C for 10 minutes for 30 cycles. To confirm that the constructed GFP fusion strains contained a diploid genome, the DNA content of the examined strains was also analysed by FACS and compared to the *S. cerevisiae* BY4741 haploid and the *S. cerevisiae* BY4743 diploid control strains of known ploidy (Fig. S1).

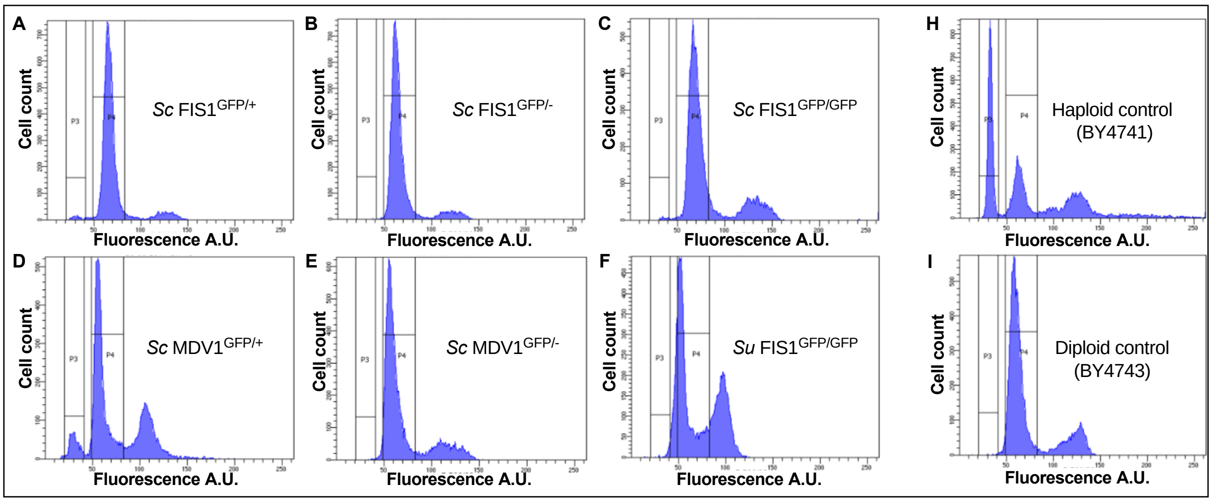

Figure S1. Analysis of ploidy in the GFP *S. cerevisiae* fusion strains. Flow cytometry analysis of the DNA content of the *FIS1*^{GFP} (A), (B), (C) and *MDV1*^{GFP} (D), (E), (F) *S. cerevisiae* diploid strains in comparison to the BY4741 (haploid control) (H) and BY4743 (diploid control) standards (I). Histograms represent SYTOX® Green fluorescence intensity (A.U.) versus cell count. BY4741 (Mean A.U. 73.7), BY4743 (Mean A.U. 82.6), *Sc* FIS1$^{GFP/+}$ (Mean A.U. 92.3), *Sc* FIS1$^{GFP/-}$ (Mean A.U. 91.8), *Sc* FIS1$^{GFP/GFP}$ (Mean A.U. 84), *Sc* MDV1$^{GFP/+}$ (Mean A.U. 86.8), *Sc* MDV1$^{GFP/-}$ (Mean A.U. 78.8), *Sc* MDV1$^{GFP/GFP}$ (Mean A.U. 97.4); A.U. represents Arbitrary Units. *Sc* represents *Saccharomyces cerevisiae*.

All strains constructed and/or manipulated in this study along with information regarding the yeast background, type of fluorophore, strain genotype, genes fused to fluorophores and fusion proteins expressed are listed in Table S1. * Indicates strains purchased from the GFP-tagged collection (Life Technologies, UK, Clone ID: YILO65C and YJL112W), ** indicates mutant strains from Yeast Deletion Collection available in the Delneri lab, *Sc* represents an *S. cerevisiae* strain background.

**Yeast organism**	**Fluorescent protein tag**	**Strain description**	**Strain description in FCS**	**Allelic state**	**Fluorescently-fused proteins**
S. cerevisiae	GFP	**S. cerevisiae* FIS1^{GFP} *MAT*a x *S. cerevisiae* FIS1^{+} *MAT*α	*Sc* FIS1$^{GFP/+}$	*FIS1*^{GFP}<br>*FIS1*$^{+}$	Fis1p-GFP
S. cerevisiae	GFP	**S. cerevisiae* FIS1^{GFP} *MAT*a x ***S. cerevisiae* ΔFIS1 *MAT*α	*Sc* FIS1$^{GFP/-}$	*FIS1*^{GFP}	Fis1p-GFP
S. cerevisiae	GFP	**S. cerevisiae* FIS1^{GFP} *MAT*a x *S. cerevisiae* FIS1^{GFP} *MAT*α	*Sc* FIS1$^{GFP/GFP}$	*FIS1*^{GFP}<br>*FIS1*^{GFP}	Fis1p-GFP<br>Fis1p-GFP
S. cerevisiae	GFP	**S. cerevisiae* MDV1^{GFP} *MAT*a x *S. cerevisiae* MDV1^{+} *MAT*α	*Sc* MDV1$^{GFP/+}$	*MDV1*^{GFP}<br>*MDV1*$^{+}$	Mdv1-GFP
S. cerevisiae	GFP	**S. cerevisiae* MDV1^{GFP} *MAT*a x<br>***S. cerevisiae* *Δ*MDV1 *MAT*α	*Sc* MDV1$^{GFP/-}$	*MDV1*^{GFP}	Mdv1-GFP
S. cerevisiae	GFP	**S. cerevisiae* MDV1^{GFP} *MAT*a x<br>*S. cerevisiae* MDV1^{GFP} *MAT*α	*Sc* MDV1$^{GFP/GFP}$	*MDV1*^{GFP}<br>*MDV1*^{GFP}	Mdv1-GFP<br>Mdv1-GFP

Table S1. Yeast library of GFP fusion strains.

Primers for quantitative RT-PCR were designed to produce an amplicon between 80–150 bp (Table S2). Optimised qPCR reactions contained 5 ng/µL of cDNA, 4 pmol each primer and 5 µL of iTAq Universal SYBR Green super Mix 2X (Bio-Rad) in a final volume of 10 µL. The amplifications were performed on a Light Cycler 480 real time System (Roche) for 35 cycles of: 15 seconds at 95°C; 30 seconds at 57°C; and 30 seconds at 72°C. Melting curve data were collected incrementing by 0.5˚C the temperature from 65˚C to 95˚C using default settings in the machine.

Primer name	Sequence (5' - 3')	Tm◦	Use in the study
MAT**a**_locus_F1	ACTCCACTTCAAGTAAGAGTTTG	58.1	Amplification of *MAT* locus for mating type switch
MATalpha_locus_F2	GCACGGAATATGGGACTACTTCG	67.3	
MAT_locus_R	AGTCACATCAAGATCGTTTATGG	61.7	
FIS1_Conf_up_F	CCCGTAGACGAGAATGCCTA	64.0	Upstream confirmation of GFP-*His3MX6* cassette insertion at the 3' end of *FIS1*
FIS1_Conf_up_GFP_R	TGACTTCAGCACGTGTCTTGT	63.6	
FIS1_Conf_dw_GFP_F	GACGGCCCTATGCTGTTATC	63.3	Downstream confirmation of GFP-His3MX6 cassette insertion at the 3' end of *FIS1*
FIS1_Conf_dw_R	CCATCTTGTCGTTTGGTCCT	63.9	
MDV1_Conf_up_F	TGCCTGGTCACAGGTTCATA	63.4	Upstream confirmation of GFP-His3MX6 cassette insertion at the 3' end of *MDV1*
MDV1_Conf_up_GFP_R	TGACTTCAGCACGTGTCTYGY	63.6	
MDV1_Conf_dw_F	GACGGCCCTATGCTGTTATC	63.3	Downstream confirmation of GFP-His3MX6 cassette insertion at the 3' end of *MDV1*
MDV1_Conf_dw_GFP_R	TTTCGACAATTTGGCATCTG	63.8	
FIS1_GFP_F	AGTCAAGGTTTAACTACGCATGG	57.0	Amplification of *FIS1*-GFP for Real-Time PCR
FIS1_GFP_R	ACTCGGCCTCTTTGTAAATGTCT	59.0	

Table S2. Primer sequences for MAT locus amplification; confirmation of GFP fusion and Real-time PCR . Tmº: Melting temperature, F: Forward, R: Reverse

	Sc FIS1$^{GFP/+}$ **Glucose**	*Sc* FIS1$^{GFP/-}$ **Glucose**	*Sc* FIS1$^{GFP/+}$ **Glycerol**	*Sc* FIS1$^{GFP/-}$ **Glycerol**	*Sc* MDV1$^{GFP/+}$ **Glucose**	*Sc* MDV1$^{GFP/-}$ **Glucose**	*Sc* MDV1$^{GFP/+}$ **Glycerol**	*Sc* MDV1$^{GFP/-}$ **Glycerol**
Number of values	107	145	103	158	108	81	131	135
Minimum	11.09	13.02	4.53	7.49	12.79	11.69	15.21	12.93
25% Percentile	16.64	20.05	16.12	15.9	16.8	16.62	25.79	26.9
Median	20.85	23.85	20.02	22.98	19.93	20.19	32.79	34.75
75% Percentile	25.66	28.04	26.56	32.94	23.59	23.7	39.9	43.75
Maximum	44.41	42.91	46.1	54.63	37.43	34.68	79.93	71.2
Mean	21.86	24.25	21.59	25.24	20.84	20.55	33.81	36.25
Std. Deviation	6.784	5.965	8.394	11.16	5.397	4.858	12.17	12.12
Std. Error of Mean	0.6559	0.4954	0.827	0.8881	0.5194	0.5398	1.064	1.043
Lower 95% CI of mean	20.56	23.28	19.95	23.49	19.81	19.47	31.71	34.19
Upper 95% CI of mean	23.16	25.23	23.23	27	21.87	21.62	35.92	38.32
95% CI of median								
Actual confidence level	96.71%	95.41%	95.18%	95.36%	95.72%	95.52%	96.44%	96.15%
Lower confidence limit	18.74	22.53	18.25	20.56	18.48	19.2	30.42	32.37
Upper confidence limit	22.04	25.15	23.03	25.76	21.35	21.62	34.9	37.09
Sum	2340	3517	2224	3988	2251	1664	4429	4894
**D'Agostino & Pearson normality test**								
K2	18.3	5.165	4.828	11.64	12.83	3.312	22.51	10.09
P value	0.0001	0.0756	0.0895	0.003	0.0016	0.1909	<0.0001	0.0064
Passed normality test (alpha=0.05)?	No	Yes	Yes	No	No	Yes	No	No
P value summary	***	ns	ns	**	**	ns	****	**
Confocal Volume (0.57fl) and Yeast volume (82fl)								
Total number of molecules per cell	**2999.47**	**3431.05**	**2880.07**	**3305.89**	**2867.12**	**2904.53**	**4717.16**	**4999.12**

Table S3. Statistical parameters and test for estimation of the total number of molecules per yeast cell [1]

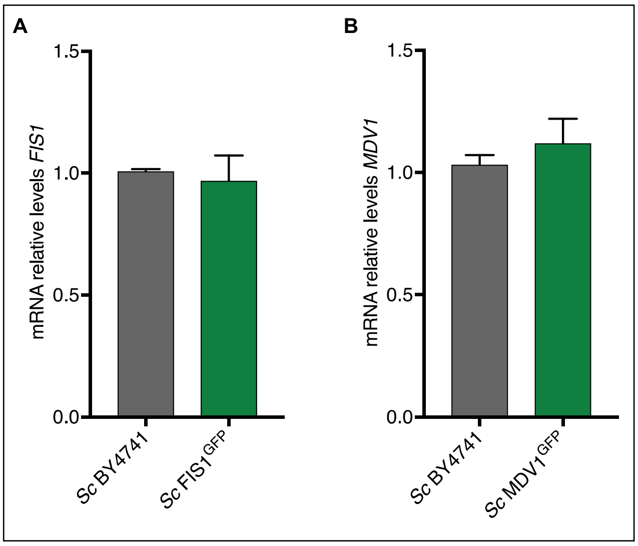

Figure S2. mRNA levels of *FIS1* and *MDV1* are not altered in GFP-tagged strains. Relative mRNA levels of (A) *FIS1* and (B) *MDV1* analysed by RT-qPCR in the wild-type and in the GFP-tagged *Sc* FIS1^{GFP} and *Sc* MDV1^{GFP} haploid strains, respectively; error bars represent standard deviation of three biological replicates; *Sc* indicates *Saccharomyces cerevisiae*. P-values from significance testing were calculated using unpaired Student's T-test.

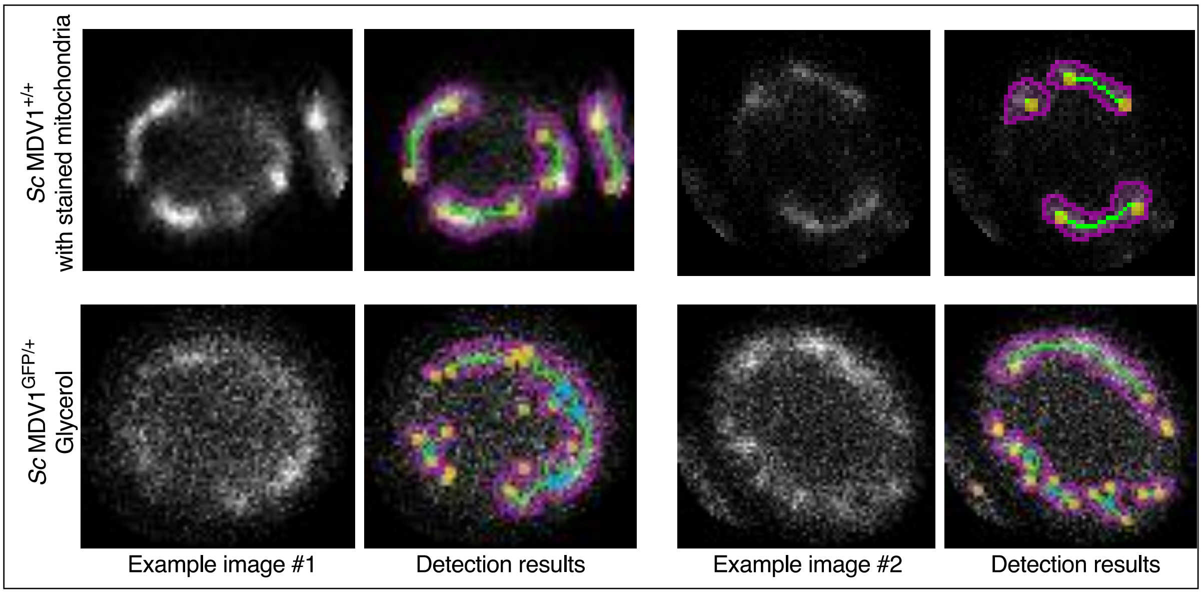

Figure S3. Quantification of cell size and mitochondrial surface in yeast. Confocal microscopy images showing: Top panel; two *Sc* MDV1$^{+/+}$ cells with stained mitochondria. Bottom panel; two *Sc* MDV1$^{GFP/+}$ cells with stained mitochondria. Coloured areas were used to quantify the size of the cell and the localisation of mitochondria in the cells. *Sc* indicates *Saccharomyces cerevisiae*. Object detection was performed using ImageJ plugin Squassh (parameters: rolling ball window = 10, regularization = 0.05, minimum intensity = 0.15, noise model = poisson and objects below 2 pixels removed).

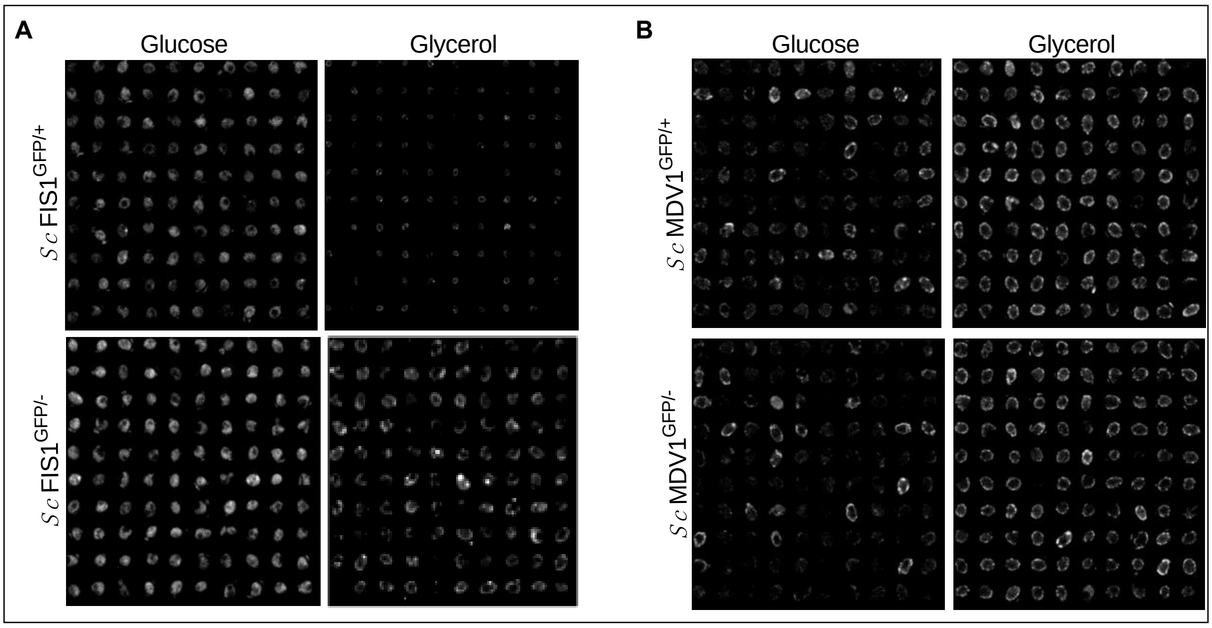

Figure S4. Characterisation of yeast cells showing mitochondrial localisation. Individual cells were segmented *and* isolated from confocal images for each strain and condition. The above images show recombined montages of 100 randomly selected cells from each individual cell image data set.

Strain name used for FCS	Growth condition	Positive cells	Negative cells
Sc $FIS1^{GFP/+}$	YP + 2 % Glucose	682	1646
Sc $FIS1^{GFP/+}$	YP + 2 % Glycerol	363	288
Sc $FIS1^{GFP/-}$	YP + 2 % Glucose	783	1265
Sc $FIS1^{GFP/-}$	YP + 2 % Glycerol	705	136
Sc $MDV1^{GFP/+}$	YP + 2 % Glucose	436	187
Sc $MDV1^{GFP/+}$	YP + 2 % Glycerol	661	50
Sc $MDV1^{GFP/-}$	YP + 2 % Glucose	440	198
Sc $MDV1^{GFP/-}$	YP + 2 % Glycerol	1042	68

Table S4. Quantification of yeast cells showing mitochondrial localisation. FCS: Fluorescence Correlation Spectroscopy; *Sc*: *Saccharomyces cerevisiae*; GFP: Green Fluorescence Protein; YP: yeast extract peptone medium

Refences

1. Jorgensen, P, et al., *Systematic identification of pathways that couple cell growth and division in yeas*t. Science, 2002. **297**(5580): p 395-400.
